# Efficacy and Safety of the “Trisection Method” Training System for Robot-Assisted Radical Cystectomy at a Single Institution in Japan

**DOI:** 10.3390/curroncol29120728

**Published:** 2022-11-29

**Authors:** Keita Nakane, Toyohiro Yamada, Risa Tomioka-Inagawa, Fumiya Sugino, Naotaka Kumada, Makoto Kawase, Shinichi Takeuchi, Kota Kawase, Daiki Kato, Manabu Takai, Koji Iinuma, Takuya Koie

**Affiliations:** Department of Urology, Gifu University Graduate School of Medicine, Gifu 5011194, Japan

**Keywords:** robot-assisted radical cystectomy, surgical training system, bladder cancer, surgical outcomes, oncological outcomes

## Abstract

To maintain a surgeon’s concentration, reduce fatigue, and train young surgeons, surgical procedures for bladder cancer are divided into the following parts: robot-assisted radical cystectomy (RARC), bowel reconstruction, and totally intracorporeal urinary diversion (ICUD) (RARC+ICUD). Each part is performed by a different surgeon (Trisection method). We retrospectively evaluated the efficacy and safety of this approach at a single institution in Japan. One hundred consecutive patients who underwent RARC+ICUD at Gifu University Hospital between November 2018 and August 2022 were included in this study. The patient background, surgical outcomes, and postoperative complications were compared between surgeries by first-, second-, and third-generation surgeons. The overall survival (OS) and recurrence-free survival (RFS) were compared between surgeries by each generation. Of the 100 patients, 19, 38, and 43 RARCs were performed by first-, second-, and third-generation surgeons, respectively. There were 35, 25, and 39 patients who underwent ileal conduit, neobladder, and ureterocutaneostomy, respectively. No significant differences were found among the patients respective to the type of ICUDs. Although the first-generation surgeon had a significantly shorter operative time with RARC, the surgical time for bowel reconstruction, length of hospital stays, and incidence of postoperative complications were not significantly different among the groups. Additionally, OS and RFS did not differ significantly among the generations. The “Trisection method” is an effective and safe concept with no difference in outcomes between the generations of surgeons.

## 1. Introduction

Radical cystectomy (RC) with pelvic lymph node dissection (PLND) and subsequent urinary diversion (UD) are the standard of care for high-risk non-muscle-invasive and muscle-invasive bladder cancer (BCa) [1]. Robotic-assisted RC (RARC) for BCa has a significantly lower transfusion rate and shorter hospital stay than open cystectomy [2,3,4]. Although the mortality rate has decreased in the era of RARC, radical cystectomy remains one of the most difficult surgeries [5,6]. Furthermore, the mortality rate is highly dependent on the number of cases presenting at a single institution per year [6]. Complications specific to RARC including peritoneal dissemination, extrapelvic lymph node metastasis, and port site recurrence, have been reported; however, their incidence has been denied in recent years [7,8]. Therefore, the surgeon’s surgical skills and institutional proficiency, including anesthesiologists, operating room nurses, clinical engineers, and other surgical staff, should be taken into consideration when selecting patients suitable for RARC.

Since 2018, after insurance in Japan began covering RARC, the number of facilities performing the procedure has gradually increased. Our institution has been performing RARC followed by total intracorporeal urinary diversion (IUCD) (RARC+ICUD) since 2018 [9,10]. As a result of the gradual increase in the number of RARCs performed worldwide, there is a trend towards shorter operative times [9,10]. Although the RARC+ICUD technique continues to improve and refine, it would be difficult for a single surgeon to perform this procedure from start to finish because of the complexity of the surgical technique and longer operative time than that in open surgery [11,12,13]. From the beginning, we followed the so-called “Trisection method”, which divides RARC+ICUD into three steps as follows: (i) RC and PLND, (ii) intestinal resection and reconstruction, and (iii) ICUD [10]. By having different surgeons perform each step, the surgeon’s concentration can be maintained, which leads to improved safety and efficiency of RARC+ICUD. Each part can be taught separately to the next generation surgeon [14,15]. Each part can be taught separately to the next generation surgeon.

We aimed to retrospectively evaluate the efficacy and safety of the “Trisection method” for RARC+ICUD in patients with BCa at a single Japanese institution.

## 2. Materials and Methods

### 2.1. Patients

This study was approved by the institutional review board of Gifu University (No: 2018-154; 18 December 2018). The requirement for informed patient consent was waived due to the retrospective nature of the study in accordance with the provisions of the Ethics Committee and Ethics Guidelines in Japan. This is because the results of the retrospective and observational studies have already been published. The details of the study can be found at https://www.med.gifu-u.ac.jp/visitors/disclosure/docs/2018-154.pdf (accessed on 11 October 2022).

From November 2018 to August 2022, 100 consecutive patients with BCa who underwent RARC+ICUD at Gifu University Hospital were enrolled in this study. The surgeons who introduced RARC+ICUD were considered first-generation surgeons, while those who were trained in the surgical techniques by the first-generation surgeons were considered second-generation surgeons. Those who were trained by first- and second-generation surgeons were considered third-generation surgeons. The clinicopathological and laboratory parameters assessed included age; sex; body mass index (BMI); clinical stage; total operative time (OT); time required for RC, to divide the bowel lumen, and to reconstruct the bowel; estimated blood loss (EBL); blood transfusion rate; incidence of intraoperative complications; urinary diversion type; time-to-fluid intake; time-to-solid intake; and postoperative length of hospital stay (LOS). The condition of pathological resection margins and the nature and frequency of postoperative complications were analyzed for each surgeon.

### 2.2. Surgical Procedure

**Figure 1:**
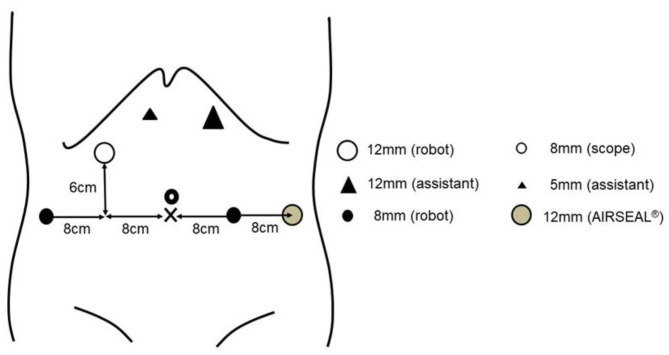
Port placements.

The surgical procedure for RARC and ICUD has been reported elsewhere in detail [9,10,16,17]. The RARC was performed in the 25° Trendelenburg position under general anesthesia using the da Vinci Xi surgical system (Intuitive Surgical, Sunnyvale, CA, USA). All procedures were performed using seven trocars (Figure 1). A 12 mm AIRSEAL^®^ port (ConMed; Utica, NY, USA) was created in the left outermost part of the abdomen. When using the SureForm™ stapler (Intuitive Surgical, Sunnyvale, CA, USA), a 12 mm trocar for the da Vinci Xi was provided in the third arm and a 6 cm cephalad to the umbilicus [10,16].

The RARC, PLND, and ICUD procedures have been previously reported [4,9,16,17,18]. The entire surgical process was divided into three parts: RARC and PLND, gastrointestinal reconstruction, and ICUD; each was performed by a different surgeon. A 60 mm, white, SureForm (Intuitive Surgical, Sunnyvale, CA, USA) was used for dissection and reconstruction of the small intestine; gastrointestinal tract reconstruction was performed by functional end-to-end anastomosis. PLND, including that of the hypogastric, external iliac, and obturator lymph nodes, was performed [10,16].

The Wallace method was used for creating an intracorporeal ileal conduit [16]. An intracorporeal ileal neobladder was created using the cross-fold U-configuration method [17]. Bilateral intracorporeal UC was performed via a retroperitoneal approach. Two small incisions were made for the UC stoma after RARC. The distal end of the ureter was then pulled out to the abdominal surface, and UC was performed according to Ariyoshi’s method [19]. Finally, the peritoneal window was closed with the ureter placed extraperitoneally.

The urinary diversion type was determined according to the surgeon’s and/or patient’s preference. Patients aged ≥80 years with advanced comorbidities and/or suspected carcinoma in the upper urinary tract were selected for UC.

### 2.3. Qualifications of the Surgeon Performing RARC

The prerequisite for a surgeon to perform RARC+ICUD was the ability to complete robot-assisted prostate surgery independently and to assist in the RARC. The first step was the dissection and reconstruction of the small intestine under the supervision of an expert surgeon. Subsequently, the surgeon performed RARC and PLND, and created an ICUD, also under the supervision.

### 2.4. Statistical Analysis

The primary endpoint was the comparison of surgical and perioperative outcomes among surgeries by first-, second-, and third-generation surgeons. The secondary endpoints were the oncological outcomes, including overall survival (OS) and recurrence-free survival (RFS), among the surgeries by three generations of surgeons. Data were analyzed using JMP 14 (SAS Institute Inc., Cary, NC, USA). Continuous variables were evaluated using the Kruskal–Wallis test and categorical variables were evaluated using Pearson’s chi-square or Fisher’s exact test. The surgical time for each procedure was defined as follows: (i) RARC: time from da Vinci roll-in to the end of RC; (ii) gastrointestinal reconstruction: time from the small intestine division to completion of functional end-to-end anastomosis; (iii) ileal conduit: time from completion of bowel reconstruction to retroperitonealization of the ileal conduit; and (iv) ileal neobladder: time from completion of bowel reconstruction to the end of new bladder wall closure. The OS was defined as the time from surgery to death due to any cause, whereas RFS was defined as the time from surgical treatment to disease progression. The Kaplan–Meier method was used to evaluate OS and RFS, and the differences were assessed according to clinical variables using the log-rank test. All *p* values were two-sided, with *p* values < 0.05 considered significant.

## 3. Results

### 3.1. Patient Characteristics

A total of 100 consecutive patients were enrolled in this study. Among these, ileal conduit construction, ileal neobladder construction, and bilateral ureterocutaneostomy were performed in 35, 25, and 39 patients, respectively. Histopathological examination of the surgical specimens revealed urothelial carcinoma in 95 patients, urachal cancer in two, and the stromal tumor of unknown malignant potential in one. The demographic data of the enrolled patients are listed in Table 1. There were no significant differences in the clinical covariates among the three groups.

The surgical and perioperative outcomes according to surgeon generation are shown in Table 2. Third-generation surgeons had significantly longer operative times (OT) than their counterparts. Intraoperative complications, resection margins, and postoperative courses were not significantly different among the three groups. Although comparisons were also performed regarding the total operative time for each generation when a different surgeon underwent the urinary diversion, no significant differences were found among the three groups.

Table 3 shows the ICUD performed according to the surgeon generation. The number of first-generation surgeons tended to be relatively small compared to the other groups. OTs according to ICUD type was not significantly different among the three groups. The gastrointestinal reconstruction median OT of third-generation surgeons (26 min) was significantly shorter than that of their counterparts (first- and second-generation surgeons, 32 min; *p* = 0.047). In this study, ureterocutaneostomy was selected for patients older than 80 years, locally advanced cases with a high risk of recurrence, patients with several comorbidities, and patients with a poor general condition. Therefore, the proportion of ureterocutaneostomy was relatively high in the present results.

According to the Clavien–Dido classification [20], the frequency of postoperative complications by surgeons of all generations is shown in Table 4. There were no significant differences among the three groups.

### 3.2. Oncological Outcomes

**Figure 2:**
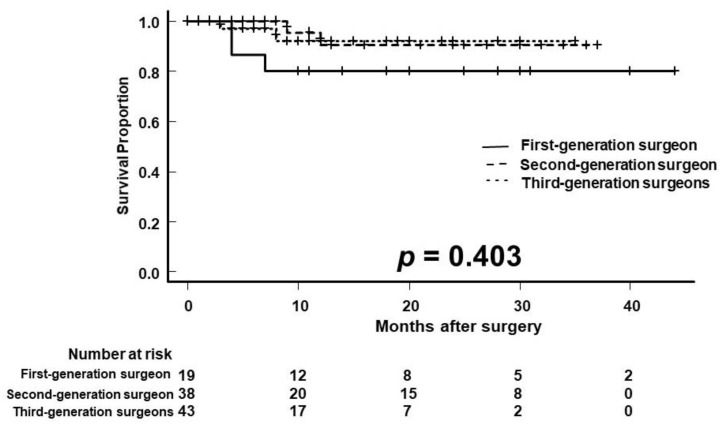
Kaplan–Meier analysis of overall survival (OS). The 1-year OS was 80.0%, 90.4%, and 92.0% in the first-, second- and third-generation surgeons, respectively.

The 1- and 2-year OS rates were 80.0% and 80.0% for first-generation surgeons, 90.4% and 90.4% for second-generation surgeons, and 92.0% and 92.0% for third-generation surgeons, respectively (Figure 2; *p* = 0.403). The median OS was not reached in any group.

**Figure 3:**
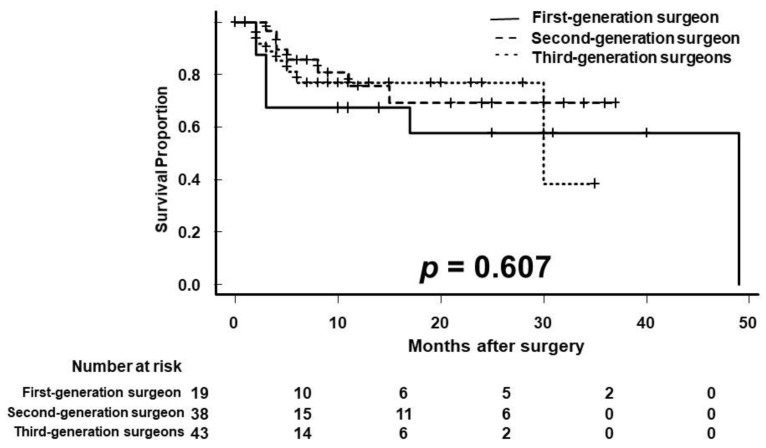
Kaplan–Meier analysis of recurrence-free survival (RFS). The 1-year RFS was 67.3%, 75.5%, and 76.8% in the first-, second-, and third-generation surgeons, respectively.

The 1- and 2-year RFS rates were 67.3% and 57.7% for first-generation surgeons, 75.5% and 69.2% for second-generation surgeons, and 76.8% and 76.8% for third-generation surgeons, respectively (Figure 3; *p* = 0.607). Although the median RFS was reached at 49 months and 30 months for first- and third-generation surgeons, it was not reached for second-generation surgeons.

## 4. Discussion

Based on the results of this study, the “Trisection method” is effective and safe for RARC+ICUD. Additionally, the results suggest that this system may facilitate the smooth transfer of surgical skills to novice surgeons, even at institutions with a small number of patients. Certainly, it is difficult to accurately assess the level of surgical technique of each generation in this study. However, the purpose of this study was not to compare which generation’s surgical technique was superior; the objective was to verify whether the RARC+ICUD technique was correctly and safely passed on from surgeons already performing it safely to the next generation of surgeons. Therefore, perioperative outcomes, oncologic outcomes, and complication rates were compared between each generation. Regarding the quality of surgery, it may be important to consider indicators such as negative resection margins and a low incidence of postoperative complications. However, there are concerns that a longer operative time may result in increased blood loss, increased cardiopulmonary burden due to the Trendelenburg position, and increased incidence of compartment syndrome. Therefore, the analysis of operative time and length of hospital stay would be meaningful as a method to assure the quality of surgery.

RARC is widely recognized as a minimally invasive procedure with lesser blood loss and shorter LOS than that in open total cystectomy [2,3,4]. However, the main problem with RARC is the longer OT than that for open surgery [2,3,4]. In recent years, ICUD has gradually been adopted worldwide [11]. However, the amount of experience required to obtain stable surgical results, especially in low-volume hospitals, remains unclear because of the complexity of the RARC+ICUD procedure. Hayn et al. conducted a study on the RARC learning curve using a statistical model [21]; it takes 21 patients to perform 6.5 h of OT and 8, 20, and 30 patients to perform 12, 16, and 20 lymph node dissections, respectively [21]. In addition, more than 30 surgeries needed to be performed to achieve a positive surgical margin rate (PSM) of <5% [21]. For cases with a pathological stage T2 or higher, a procedural experience of 24 or more was required to achieve a PSM of <15% [21]. Wijburg et al. reported that 75 surgeries needed to be performed to reach a plateau in OT, 88 for EBL, and 137 for the reduction in the frequency of serious complications [22]. Lopez et al. divided 62 RARC patients with ICUD into two surgeons; the first 20 cases were treated by surgeons with experience in robotic surgery and the remaining by junior surgeons. There was no difference in the perioperative outcomes [23]. In a single-center, single-surgeon study of RARC followed by intracorporeal ileal neobladder, 137 consecutive patients were divided into three groups, and the perioperative outcomes and complications were compared [24]. This study reported that the group in which the surgery was most recently performed had a shorter mean OT and LOS, and a lower complication rate [24]. Based on these reports, it appears that a minimum of 20 surgeries performed under the supervision of a skilled surgeon are necessary to safely perform RARC. We believe that it is important for novice surgeons performing RARC to have a safe and effective experience with the initial cases until they become proficient. Therefore, our “Trisection method” may be an effective means to obtain safe and stable surgical results.

ICUD was considered a technically challenging procedure by several urologists compared with extracorporeal UD (ECUD) because of the perceived complexity of bowel manipulation [25]. ICUD has a steep learning curve, and a prolonged anesthesia and surgery time [26]. Several surgeons initially adopted ECUDs and hybrid approaches to continuously improve their surgical skills and expertise [25]; only a small number of surgeons performed ICUD [25]. Since then, the number of facilities that have adopted ICUD has gradually increased [11,25]. According to the International Robotic Cystectomy Consortium (IRCC) outcomes of 1,094 patients who underwent RARC+ICUD, the use of RARC+ICUD increased from 9% in 2005 to 97% in 2016 [11]. The IRCC also demonstrated that RARC+ICUD had a shorter OT and lower EBL than RARC+ECUD [24]. RARC+ICUD is associated with a significantly lower EBL, intraoperative transfusion rate, 90-day rehospitalization rate, surgery-related complications, and shorter LOS than open RC [26]. A head-to-head single-center prospective study by two expert surgeons found that ICUD and ECUD for the ileal conduit construction had comparable perioperative outcomes and complication rates after RARC [27]. Tan et al. reported the perioperative outcomes and complications after the transition from ECUD to ICUD following RARC [28]. For the ileal conduit, ICUD was statistically associated with lower median OT, EBL, and 30-day perioperative complication rates when compared to ECUD [26]. Collins et al. compared the surgical outcomes of 67 patients who underwent RARC with an intracorporeal neobladder construction between experienced and inexperienced surgeons [14]. They concluded that in institutions with experienced robotic teams, the learning curve of new surgeons was affected; shorter OTs and lower conversion and complication rates were obtained in the initial RARC by inexperienced surgeons [14]. In our results, third-generation surgeons tended to have shorter OTs for urinary diversion, although the difference was not statistically significant. According to PLND, there were no significant differences between generations even though the median number of lymph nodes dissected tended to increase gradually with each generation. Appropriate surgical education by experts is considered important for successful ICUD.

Dell’Oglio et al. advocated a systematic education for RARC+ICUD, starting with an e-learning module, followed by 5 days of preclinical simulation-based training, 10 steps of clinical modular training [29]. Although this training model has not yet been validated, it was developed with opinions from RARC experts, and its effectiveness may need to be further tested in the future. We did not adopt a highly systematic educational approach as prescribed by the European Association of Urology Robotic Urology Section (ERUS). However, from a safety perspective, we require that new surgeons are able to safely complete a robotic-assisted radical prostatectomy and assist a RARC with an understanding of the surgical procedure as a minimum. In this study, the perioperative outcomes obtained with our “Trisection method” showed that second- and third-generation surgeons did not have inferior surgical outcomes compared to first-generation surgeons. However, since we have not established an objective evaluation method, such as the educational protocol proposed by ERUS, we cannot deny the possibility that surgical outcomes may vary depending on the subjectivity of the instructor’s evaluation of the surgeon.

Our study had several limitations. First, the retrospective design of this study may have introduced a bias. Second, compared with high-volume centers worldwide, the number of cases per facility is small in Japan. Therefore, the number of cases that one urologist can experience is limited. If one surgeon performed more surgeries, it is possible that the results would be better than the surgical outcomes of this study. In addition, it seems necessary to establish an educational system for RARC+ICUD, especially in Japan. Third, the relatively short follow-up period may have been insufficient to examine oncological outcomes. Finally, the number of patients in whom ureterocutaneostomy was selected as a urinary diversion option was relatively high compared to other options. This could be attributed to the fact that the study enrolled a larger number of elderly patients with comorbidities.

## 5. Conclusions

The “Trisection method” in our institution may be an effective and safe training system for RARC+ICUD, and it may be possible to be replicated even at institutions with a relatively small number of RARC+ICUD cases.

## Figures and Tables

**Table 1:** Patient characteristics.

Clinical Covariates	Total	First-Generation Surgeon	Second-Generation Surgeon	Third-Generation Surgeons	*p*-Value
Number	100	19	38	43	
Age, year [median (IQR)]	73.0 (67.0–78.0)	73.0 (65.5–78.5)	74.0 (67.2–78.0)	73.0 (68.5–78.0)	0.900
Gender [number (%)]					0.085
Male	78 (78.0)	14 (73.7)	26 (68.4)	38 (88.4)	
Female	22 (22.0)	5 (26.3)	12 (31.6)	5 (11.6)	
BMI, kg/m^2^ [median (IQR)]	23.1 (20.2–25.2)	24.0 (19.9–26.6)	21.8 (19.8–24.1)	24.1 (21.9–25.6)	0.070
Clinical T-stage [number (%)]					0.374
T0	7 (7.0)	1 (5.3)	3 (7.9)	3 (7.1)	
Tis	3 (3.0)	1 (5.3)	1 (2.6)	1 (2.4)	
T1	7 (7.0)	1 (5.3)	1 (2.6)	5 (11.9)	
T2	43 (43.0)	4 (21.1)	17 (44.7)	22 (52.4)	
T3	27 (27.0)	8 (42.1)	11 (28.9)	8 (19.0)	
T4	12 (12.0)	4 (21.1)	5 (13.2)	3 (7.1)	
Tx	1 (1.0)	1 (5.3)	0 (0.0)	0 (0.0)	
Clinical N-stage, number (%)					0.813
negative	90 (90.0)	16 (84.2)	34 (89.5)	40 (93.0)	
positive	10 (10.0)	3 (15.8)	4 (10.5)	3 (7.0)	
Clinical M-stage, number (%)					0.512
M0	99 (99.0)	19 (100.0)	38 (100.0)	42 (97.7)	
M1	1 (1.0)	0 (0.0)	0 (0.0)	1 (2.3)	
Neoadjuvant chemotherapy, number (%)	69 (69.0)	14 (73.7)	27 (71.1)	28 (65.1)	0.751
Follow-up period, months [median (IQR)]	9.0 (3.0–24.0)	14.0 (4.0–29.0)	11.5 (4.0–25.0)	6.0 (2.5–14.0)	0.105

IQR, interquartile range; BMI, body mass index.

**Table 2:** Surgical and perioperative outcomes.

Clinical Characteristics	Total	First-Generation Surgeon	Second-Generation Surgeon	Third-Generation Surgeon	*p*-Value
Operative time, minutes [median (IQR)]	398.5 (296.7–484.2)	457.0 (319.0–484.5)	389.5 (281.7–461.2)	400.0 (319.0–483.5)	0.838
Time for cystectomy, minutes [median (IQR)]	122.0 (100.5–142.0)	106.0 (96.0–133.0)	110.0(98.0–134.2)	130.5 (115.0–151.7)	0.019
Estimated blood loss, mL [median (IQR)]	205.0 (100.0–396.2)	240.0 (105.0–392.5)	150.0(96.2–261.2)	320.0 (137.5–420.0)	0.137
Blood transfusion [number (%)]	8 (8.0)	0 (0.0)	3 (7.9)	5 (11.6)	0.298
Intraoperativecomplications [number (%)]	6 (6.0)	2 (10.5)	1 (2.6)	3 (7.0)	0.466
Pathological T stage [number, %]					0.079
pT0	19 (19.6)	4 (21.1)	8 (21.1)	7 (17.5)	
pT1	8 (8.2)	0	3 (7.9)	5 (12.5)	
pT2	20 (20.6)	3 (15.8)	7 (18.4)	10 (25.0)	
pT3	31 (32.0)	3 (15.8)	14 (36.8)	14 (35.0)	
pT4	10 (10.3)	6 (31.6)	3 (7.9)	1 (2.5)	
pTa/pTis	9 (9.2)	3 (15.8)	3 (7.9)	3 (7.9)	
Pathological N stage [number, %]					0.191
pN0	35 (35.7)	6 (31.6)	10 (26.3)	19 (46.3)	
pN1	5 (5.1)	0 (0.0)	4 (10.5)	1 (2.4)	
pN2	2 (2.0)	1 (5.3)	0 (0.0)	1 (2.4)	
pNx	56 (57.1)	12 (63.2)	24 (63.2)	20 (48.8)	
Surgical margin [number (%)]					0.140
RM0	95 (95.0)	16 (84.2)	38 (100.0)	41 (95.3)	
RM1	2 (2.0)	1 (5.3)	0 (0.0)	1 (2.3)	
RMx	3 (3.0)	2 (10.5)	0 (0.0)	1 (2.3)	
Time-to-liquid intake, days [median (IQR)]	1.0 (1.0–1.0)	1.0 (1.0–1.0)	1.0 (1.0–1.0)	1.0 (1.0–1.0)	>0.999
Time-to-solid intake, days [median (IQR)]	3.0 (2.0–3.0)	3.0 (2.0–3.0)	2.0 (2.0–3.0)	3.0 (2.0–4.0)	0.495
LOS, days [median (IQR)]	18.0 (15.0–22.0)	19.0 (16.5–21.0)	18.5 (14.0–21.7)	18.0 (15.0–22.5)	0.583

IQR, interquartile range; RM, resection margin; LOS, Length of hospital stay.

**Table 3:** Surgical outcomes according to the intracorporeal urinary diversion.

	Total	First-Generation Surgeon	Second-Generation Surgeon	Third-Generation Surgeons	*p*-Value
Number	100	25	37	37	
Urinary diversion type [number (%)]					
Ileal conduit	35 (35.0)	8 (32.0)	10 (27.0)	17 (45.9)	0.031
Neobladder	25 (25.0)	11 (44.0)	10 (27.0)	4 (10.8)	
Ureterocutaneostomy	39 (39.0)	6 (24.0)	17 (45.9)	16 (43.2)	
Urinary diversion operative time, minutes [median (interquartile range)]					
Ileal conduit	104.0 (91.0–120.0)	106.5 (88.7–126.7)	106.5 (93.7–118.5)	100.0 (94.0–114.0)	0.961
Neobladder	182.0 (159.0–231.0)	182.0 (165.5–218.5)	219.0 (162.0–262.5)	169.5 (153.0–185.0)	0.421
Ureterocutaneostomy	28.0 (20.0–36.7)	26.0 (25.2–29.0)	28.0 (22.2–31.5)	28.5 (20.0–48.5)	0.829

**Table 4:** Postoperative complications according to the Clavien–Dindo classification.

Complication [Number (%)]	Total	First-Generation Surgeon	Second-Generation Surgeon	Third-Generation Surgeons	*p*-Value
Any grade					
Ileus	16 (16.0)	2 (10.5)	7 (18.4)	7 (16.7)	0.742
Pyelonephritis	24 (24.0)	3 (15.8)	11 (28.9)	10 (23.3)	0.542
Sepsis	4 (4.0)	1 (5.3)	1 (2.6)	2 (4.7)	0.856
Pelvic abscess	3 (3.0)	0	1 (2.6)	2 (4.7)	0.604
Surgical site infection	2 (2.0)	0	0	2 (4.7)	0.259
Lymphorrhea	1 (1.0)	1 (5.3)	0	0	0.116
Cardiac disorder	2 (2.0)	0	1 (2.6)	1 (2.3)	0.783
Anastomotic leakage	2 (2.0)	1 (5.3)	1 (2.6)	0	0.370
Anastomotic stricture	4 (4.0)	0	1 (2.6)	3 (7.0)	0.374
Grade ≥3					
Anastomotic stricture	4 (4.0)	0	1 (2.6)	3 (7.0)	0.374
Pelvic abscess	2 (2.0)	0	1 (2.6)	1 (2.3)	0.783
Sepsis	2 (2.0)	0	1 (2.6)	1 (2.3)	0.783
Anastomotic leakage	1 (1.0)	0	1 (2.6)	0	0.370
Surgical site infection	1 (1.0)	0	0	1 (2.3)	0.512
Lymphorrhea	1 (1.0)	1 (5.3)	0	0	0.116

## Data Availability

Data and material are provided in this paper.

## References

[1] Witjes J.A., Bruins H.M., Cathomas R., Compérat E.M., Cowan N.C., Gakis G., Hernández V., Linares Espinós E., Lorch A., Neuzillet Y. (2021). European Association of Urology Guidelines on Muscle-invasive and Metastatic Bladder Cancer: Summary of the 2020 Guidelines. Eur. Urol..

[2] Parekh D.J., Reis I.M., Castle E.P., Gonzalgo M.L., Woods M.E., Svatek R.S., Weizer A.Z., Konety B.R., Tollefson M., Krupski T.L. (2018). Robot-assisted radical cystectomy versus open radical cystectomy in patients with bladder cancer (RAZOR): An open-label, randomised, phase 3, non-inferiority trial. Lancet.

[3] Bochner B.H., Dalbagni G., Marzouk K.H., Sjoberg D.D., Lee J., Donat S.M., Coleman J.A., Vickers A., Herr H.W., Laudone V.P. (2018). Randomized Trial Comparing Open Radical Cystectomy and Robot-assisted Laparoscopic Radical Cystectomy: Oncologic Outcomes. Eur. Urol..

[4] Sathianathen N.J., Kalapara A., Frydenberg M., Lawrentschuk N., Weight C.J., Parekh D., Konety B.R. (2019). Robotic Assisted Radical Cystectomy vs Open Radical Cystectomy: Systematic Review and Meta-Analysis. J. Urol..

[5] Bochner B.H., Dalbagni G., Sjoberg D.D., Silberstein J., Keren Paz G.E., Donat S.M., Coleman J.A., Mathew S., Vickers A., Schnorr G.C. (2015). Comparing Open Radical Cystectomy and Robot-assisted Laparoscopic Radical Cystectomy: A Randomized Clinical Trial. Eur. Urol..

[6] Zakaria A.S., Santos F., Dragomir A., Tanguay S., Kassouf W., Aprikian A.G. (2014). Postoperative mortality and complications after radical cystectomy for bladder cancer in Quebec: A population-based analysis during the years 2000–2009. Can Urol. Assoc. J..

[7] Nguyen D.P., Al Hussein Al Awamlh B., Wu X., O’Malley P., Inoyatov I.M., Ayangbesan A., Faltas B.M., Christos P.J., Scherr D.S. (2015). Recurrence patterns after open and robot-assisted radical cystectomy for bladder cancer. Eur. Urol..

[8] Elsayed A.S., Gibson S., Jing Z., Wijburg C., Wagner A.A., Mottrie A., Dasgupta P., Peabody J., Hussein A.A., Guru K.A. (2021). Rates and Patterns of Recurrences and Survival Outcomes after Robot-Assisted Radical Cystectomy: Results from the International Robotic Cystectomy Consortium. J. Urol..

[9] Koie T., Ohyama C., Makiyama K., Shimazui T., Miyagawa T., Mizutani K., Tsuchiya T., Kato T., Nakake K. (2019). Utility of robot-assisted radical cystectomy with intracorporeal urinary diversion for muscle-invasive bladder cancer. Int. J. Urol..

[10] Nakane K., Enomoto T., Tomioka M., Taniguchi T., Kawase M., Kato D., Takai M., Iinuma K., Seike K., Hagiwara N. (2021). Favorable surgical outcomes and perioperative complication rates after robotic radical cystectomy and intracorporeal urinary diversion at a single, low-volume center: Initial experience with 65 consecutive cases. World J. Clin. Surg..

[11] Hussein A.A., May P.R., Jing Z., Ahmed Y.E., Wijburg C.J., Canda A.E., Dasgupta P., Shamim Khan M., Menon M., Peabody J.O. (2018). Outcomes of Intracorporeal Urinary Diversion after Robot-Assisted Radical Cystectomy: Results from the International Robotic Cystectomy Consortium. J. Urol..

[12] Hussein A.A., Dibaj S., Hinata N., Field E., O’leary K., Kuvshinoff B., Mohler J.L., Wilding G., Guru K.A. (2016). Development and Validation of a Quality Assurance Score for Robot-assisted Radical Cystectomy: A 10-year Analysis. Urology.

[13] Mastroianni R., Ferriero M., Tuderti G., Anceschi U., Bove A.M., Brassetti A., Misuraca L., Zampa A., Torregiani G., Ghiani E. (2022). Open Radical Cystectomy versus Robot-Assisted Radical Cystectomy with Intracorporeal Urinary Diversion: Early Outcomes of a Single-Center Randomized Controlled Trial. J. Urol..

[14] Collins J.W., Tyritzis S., Nyberg T., Schumacher M.C., Laurin O., Adding C., Jonsson M., Khazaeli D., Steineck G., Wiklund P. (2014). Robot-assisted radical cystectomy (RARC) with intracorporeal neobladder—What is the effect of the learning curve on outcomes?. BJU Int..

[15] Filson C.P., Tan H.J., Chamie K., Laviana A.A., Hu J.C. (2016). Determinants of radical cystectomy operative time. Urol. Oncol..

[16] Nakane K., Muramatsu Maekawa Y., Iinuma K., Mizutani K., Makiyama K., Koie T. (2019). Utility technique of a totally intracorporeal ileal conduit after robot-assisted radical cystectomy. Int. J. Urol..

[17] Koie T., Ohyama C., Yoneyama T., Nagasaka H., Yamamoto H., Imai A., Hatakeyama S., Hashimoto Y. (2018). Robotic cross-folded U-configuration intracorporeal ileal neobladder for muscle-invasive bladder cancer: Initial experience and functional outcomes. Int. J. Med. Robot..

[18] Koie T., Ohyama C., Yamamoto H., Imai A., Hatakeyama S., Yoneyama T., Hashimoto Y., Yoneyama T., Tobisawa Y., Yamauchi A. (2017). The feasibility and effectiveness of robot-assisted radical cystectomy after neoadjuvant chemotherapy in patients with muscle-invasive bladder cancer. Jpn. J Clin Oncol..

[19] Ariyoshi A., Fusijawa Y., Ohshima K., Hiratsuka Y., Sakamoto K. (1975). Catheterless cutaneous ureterostomy. J. Urol..

[20] Dindo D., Demartines N., Clavien P.A. (2004). Classification of surgical complications: A new proposal with evaluation in a cohort of 6336 patients and results of a survey. Ann. Surg..

[21] Hayn M.H., Hussain A., Mansour A.M., Andrews P.E., Carpentier P., Castle E., Dasgupta P., Rimington P., Thomas R., Khan S. (2010). Learning curve of robot-assisted radical cystectomy: Results from the International Robotic Cystectomy Consortium. Eur. Urol..

[22] Wijburg C.J., Hannink G., Michels C.T.J., Weijerman P.C., Issa R., Tay A., Decaestecker K., Wiklund P., Hosseini A., Sridhar A. (2022). Learning curve analysis for intracorporeal robot-assisted radical cystectomy: Results from the EAU Robotic Urology Section Scientific Working Group. Eur. Urol. Open Sci..

[23] López-Molina C., Carrion A., Campistol M., Piñero A., Lozano F., Salvador C., Raventós C.X., Trilla E. (2022). Evaluating the impact of the learning curve on the perioperative outcomes of robot-assisted radical cystectomy with intracorporeal urinary diversion. Actas Urol. Esp..

[24] Tuderti G., Mastroianni R., Brassetti A., Bove A.M., Misuraca L., Anceschi U., Ferriero M., Gallucci M., Simone G. (2021). Robot-assisted radical cystectomy with intracorporeal neobladder: Impact of learning curve and long-term assessment of functional outcomes. Minerva Urol. Nephrol..

[25] Tanneru K., Jazayeri S.B., Kumar J., Alam M.U., Norez D., Nguyen S., Bazargani S., Ganapathi H.P., Bandyk M., Marino R. (2021). Intracorporeal versus extracorporeal urinary diversion following robot-assisted radical cystectomy: A meta-analysis, cumulative analysis, and systematic review. J. Robot. Surg..

[26] Mortezavi A., Crippa A., Kotopouli M.I., Akre O., Wiklund P., Hosseini A. (2022). Association of Open vs Robot-Assisted Radical Cystectomy With Mortality and Perioperative Outcomes Among Patients With Bladder Cancer in Sweden. JAMA Netw. Open..

[27] Bertolo R., Agudelo J., Garisto J., Armanyous S., Fergany A., Kaouk J. (2019). Perioperative Outcomes and Complications after Robotic Radical Cystectomy With Intracorporeal or Extracorporeal Ileal Conduit Urinary Diversion: Head-to-head Comparison From a Single-Institutional Prospective Study. Urology.

[28] Tan T.W., Nair R., Saad S., Thurairaja R., Khan M.S. (2019). Safe transition from extracorporeal to intracorporeal urinary diversion following robot-assisted cystectomy: A recipe for reducing operative time, blood loss and complication rates. World J. Urol..

[29] Dell’Oglio P., Turri F., Larcher A., D’Hondt F., Sanchez-Salas R., Bochner B., Palou J., Weston R., Hosseini A., Canda A.E. (2022). Definition of a Structured Training Curriculum for Robot-assisted Radical Cystectomy with Intracorporeal Ileal Conduit in Male Patients: A Delphi Consensus Study Led by the ERUS Educational Board. Eur. Urol. Focus..

